# Evolution of educational inequalities in life and health expectancies at 25 years in Belgium between 2001 and 2011: a census-based study

**DOI:** 10.1186/s13690-019-0330-8

**Published:** 2019-02-14

**Authors:** Françoise Renard, Brecht Devleesschauwer, Herman Van Oyen, Sylvie Gadeyne, Patrick Deboosere

**Affiliations:** 1 Department of Epidemiology and public health, Sciensano, Rue Juliette Wytsmanstraat 14, 1050 Brussels, Belgium; 2 Department of Veterinary Public Health and Food Safety, Ghent University, Merelbeke, Belgium; 3 Department of Public Health and Primary Care, Ghent University, Ghent, Belgium; 4 Interface Demography, Section Social Research, Vrije Universiteit Brussels, Brussels, Belgium

**Keywords:** Health inequality, Socio-economic inequality, Life expectancy, Health expectancy, Disability-free life expectancy, Belgium, Trends

## Abstract

**Background:**

Reducing socio-economic health inequalities is a public health priority, necessitating careful monitoring that should take into account changes in the population composition. We analyzed the evolution of educational inequalities in life expectancy and disability-free life expectancy at age 25 (LE_25_ and DFLE_25_) in Belgium between 2001 and 2011.

**Methods:**

The 2001 and 2011 census data were linked with the national register data for a five-year mortality follow up. Disability prevalence estimates from the health interview surveys (2001 to 2013) were used to compute DFLE according to Sullivan’s method. LE_25_ and DFLE_25_ were computed by educational level (EL). Absolute differentials of LE_25_ and DFLE_25_ were calculated for each EL and for each period, as well as composite inequality indices (CII) of population-level impact of inequality. Changes over the 10-year period were then calculated for each inequality index.

**Results:**

The LE_25_ increased in all ELs and both genders, except in the lowest EL for women. The increase was larger in the highest EL, leading in 2011 to 6.07 and 4.58 years for the low-versus-high LE_25_ gaps respectively in men and women, compared to 5.19 and 3.76 in 2001, namely 17 and 22% increases. The upwards shift of the EL distribution led to a limited 7% increase of the CII among men but no change in women.

The substantial increase of the DFLE_25_ in males with high EL (+ 4.5 years) and the decrease of the DFLE_25_ in women with low EL, results in a substantial increase of all considered DFLE_25_ inequality measures in both genders. In 2011, DFLE_25_ gaps were respectively 10.4 and 13.5 years in males and females compared to 6.51 and 9.30 in 2001, representing increases of 61 and 44% for the gaps, and 72 and 20% for the CII.

**Conclusion:**

The LE_25_ increased in all ELs, but at a higher pace in highly educated, leading to an increase in the LE_25_ gaps in both genders. After accounting for the upwards shift of the educational distribution, the population-level inequality index increased only for men. The DFLE_25_ increased only in highly educated men, and decreased in low educated women, leading to large increases of inequalities in both genders. A general plan to tackle health inequality should be set up, with particular efforts to improve the health of the low educated women.

**Electronic supplementary material:**

The online version of this article (10.1186/s13690-019-0330-8) contains supplementary material, which is available to authorized users.

## Introduction

Socioeconomic (SE) gradients in health outcomes, also referred to as SE health inequalities, are a consistent finding in public health epidemiologic research [1–4]. Reducing health inequalities is a crucial public policy issue, at the crossroad of health, social and economic policies [5], and has led to international and (sub)national commitments [6–11]. Such policies require sustained actions in many policy areas, including addressing labor market and working conditions, comprehensive strategies to improve health habits, universal access to health care and education [12, 13]. A careful monitoring is essential to pilot and assess policies aiming at inequality reduction [14].

Accumulated evidence showed that inequalities in mortality persisted over the last decades across European countries [15]. Describing how SE inequalities change over time – that is determining the direction and the magnitude of the change – is however challenging. Firstly, different inequality indicators (e.g., absolute versus relative measures) may result in opposite conclusions [16–18]. It is therefore recommended to use multiple inequality indices rather than a single one when assessing trends [19–21]. Secondly, shifts can occur over time in the SE composition of the population, complicating the interpretation of the observed trends in health inequalities, changes in group sizes leading to a different population-level impact of inequalities [22]. The assessment of the population-level impact therefore requires more complex indices [19]. Finally, the change in inequality should always be interpreted along with the evolution of the health outcomes: an improvement of health that benefits all SE groups, including the lowest ones, is a valuable outcome regardless of whether inequalities decrease or not [16, 17], while a decrease of inequalities associated with a worsening of health would be unethical [23]. Also, improvement in the SE distribution is a valuable outcome [24].

In Belgium, the study of inequalities in mortality, life expectancy and health expectancy really started in the early 2000s with the availability of data from the nineties [24–29]. This was made possible thanks to the construction of the ‘National Mortality Database’, which links the successive censuses to the National Register to allow for a mortality and emigration follow up [30]. Since censuses occur every 10 years, and a few years are needed to ensure the follow up, the construction of the linked database for the census 2011 could only be achieved in 2018.

The aim of this contribution is to determine the size of the inequalities in life expectancy (LE) and disability-free LE (DFLE) by educational level in Belgium in 2011, and to compare these inequalities to the ones observed 10 years earlier, in 2001. Firstly, we describe the absolute inequalities in LE and DFLE at different key ages in 2011. Secondly, we examine if LE and DFLE at age 25 (LE_25_, DFLE_25_) by educational level have changed between 2001 and 2011. Thirdly, we investigate if the educational differences in LE_25_ and DFLE_25_ have changed over the same period. Finally, we compute summary measures of inequality and examine if the results can indeed be interpreted as changes in inequality.

## Methods

### Data

To calculate socioeconomic (SE) inequalities in LE and DFLE, data on mortality and health by SE status (SES) are needed.

Mortality data by SES were derived from an individual linkage of the 2001 and 2011 censuses with data from the National Register, including information on vital status and emigration during five years of follow up [25, 29].

The prevalence of the health status by SES was obtained from the Belgian Health Interview Surveys (HIS) [31]. The HIS contains the questions of the Minimum European Health Module [32], with items on self-rated health, chronic diseases, and health-related limitations in daily activities. The health status indicator chosen in this study is the Global Activity Limitation Indicator (GALI) [33], allowing for the computation of the health expectancy indicator called “Disability-Free Life Expectancy” or “Healthy Life Years” [34]. Data from the HIS 2001 and 2004 were pooled to estimate the health status prevalence that will be applied to the 2001 census (since the indicator was not available in the HIS 1997) data from the HIS 2008 and 2013 were pooled to estimate the prevalence applied to the 2011 census.

### Socio-economic position

SE position was measured using the highest level of educational attainment obtained by the individual. The census and the HIS use the same educational categories. Education was classified according to the International Standard Classification of Education [35]. The categories ‘No schooling’, ‘Primary’ and ‘Lower secondary education’ (ISCED 0, 1, 2) were pooled and classified as ‘Low’; the categories ‘Upper secondary’ and ‘Post-secondary no-tertiary education’ (ISCED 3, 4) were pooled into the ‘Mid’ class, and the categories of tertiary education (ISCED 5, 6) were classified as ‘High’.

The 2001 census was semi-administrative, with most of the SE variables collected through a mandatory SE postal survey organized by the Federal Public Service Economy [36]. The 2011 census was fully administrative. The educational variable was constructed by updating the 2001 educational level registered in the 2001 census with administrative data on new graduates, originating from the (regional) ministries of education. For this reason, the 2011 educational level was unknown for the new migrants. We therefore focused our analysis on people born in Belgium. The study population thus consisted of people registered in the Belgian National Register, born in Belgium and aged 25 years and over at the time of the census. In the main scenario, published in the core of the manuscript, we focused on inequalities among people with a known educational attainment, ignoring the missing values for education. Besides this main scenario, we conducted alternative analyses in which we grouped the missing values for educational attainment together with the lowest educational level (see Additional file 1: Appendix).

**Fig. 1:**
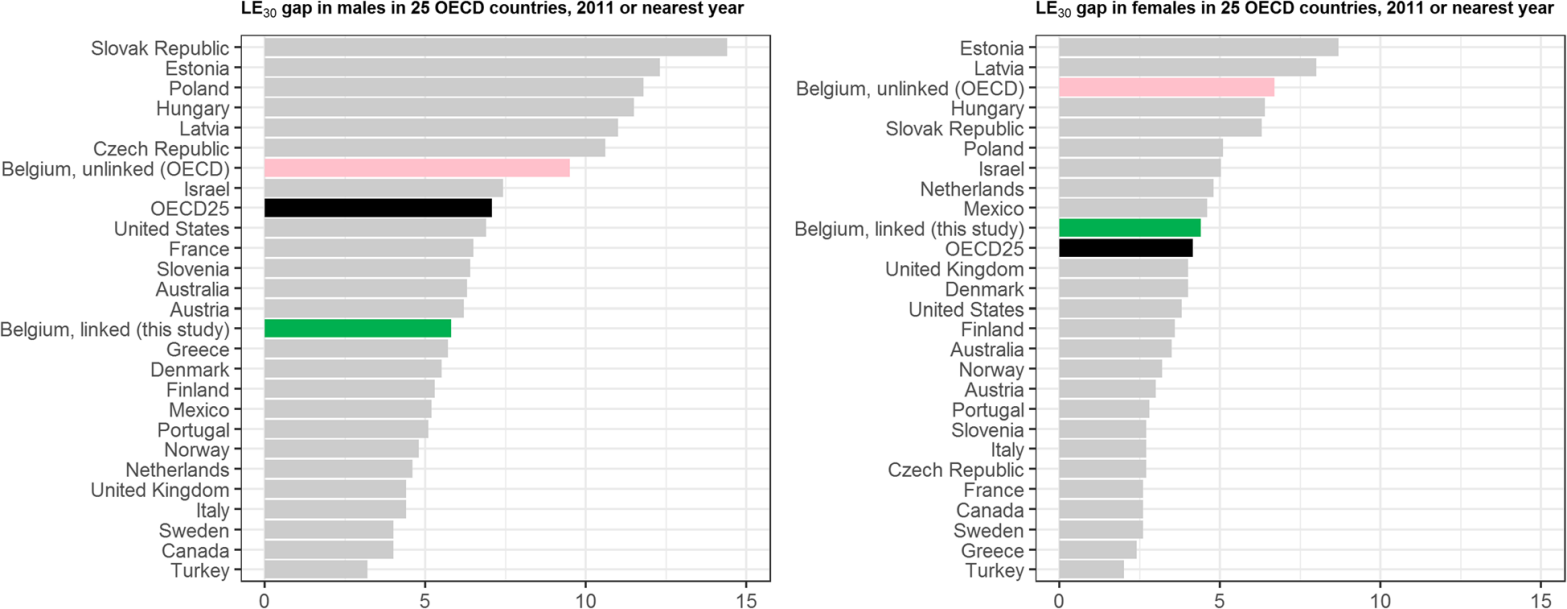
Change in the ranking of Belgium in Life Expectancy gap between the lowest and the highest educational level at age 30, after using linked data for Belgium, 2011. Data originate from OECD [37], except the linked data for BE that come from the present study

### Health outcomes

We calculated LE and DFLE by sex and educational attainment at age 25, 30, 50, 65 years because those specific ages are regularly chosen in international comparisons [3–5, 37]. As formal education is mostly completed in young adulthood, we did not consider younger ages.

For the computation of LE, abridged life tables with five-year age groups were constructed, with a last open interval at age 85. The age-specific mortality rates were computed using Lexis expansion, to account for the ageing of population during follow up [38]. The DFLE and its variance were computed using Sullivan’s method [39]. The DFLE variance was calculated as the sum of the disability prevalence variance and the mortality variance.

### Inequality indicators

#### Absolute differences in health outcomes

For assessing the evolution of the inequalities in LE, DFLE, we focused on age 25 to be in line with previous works and a recent OECD study [5].

To investigate the evolution of gaps (absolute inequality) in LE and DFLE, absolute differences in LE_25_ and DFLE_25_ (in years) between each EL and the highest EL and changes therein between 2001 and 2011 were calculated.

The statistical significance of the difference between two LE or DFLE either over time (evolution of the EL-specific health status), or between educational levels (inequality), was conservatively tested by the following Z-score [39]:

Z (difference in (DF)LE) = $\frac{(DF) LE(1)-(DF) LE(2)}{\ \sqrt{S^2(DF) LE(1)+{S}^2(DF) LE(2).}}$

(DF)LE: (Disability-Free) Life Expectancy; 1 and 2 refers to the two (DF)LE to compare

S^2^(DF)LE: variance on (Disability-Free) Life Expectancy

#### Summary measures of socio-economic inequalities

As educational distributions change over time, the evaluation of the widening or narrowing of the life expectancy gap between educational groups should address changes in the educational distribution in the population. Therefore, we used the composite index of inequality (CII) as an overall population measure of inequality [24]. The absolute CII (*CII*_*abs*_) is the sum of the weighted (*w*_*i*_) difference in the LE (DFLE) between each educational group (*i*) and the tertiary education category, with *w*_*i*_ proportional to the size of educational categories:

$$ {CII}_{abs}=\sum \limits_i\left[(DF)L{E}_{Highest}-(DF)L{E}_i\right]\ast {w}_i $$

The relative CII (*CII*_*rel*_) is the *CII*_*abs*_ divided by the overall population’s LE (DFLE) and is interpreted as the percentage change in the overall LE (DFLE) that would occur if all socio-economic groups had the LE (DFLE) of the population with tertiary education.

$$ {CII}_{rel}=\frac{CII_{abs}}{(DF) LE\  whole\ population} $$

The variance of the *CII*_*abs*_ and *CII*_*rel*_ was calculated using a Monte Carlo approach [40].

Changes in *CII*_*abs*_ were calculated as the absolute difference between the 2011 and the 2001 *CII*_*abs*_, and changes in *CII*_*rel*_ as the absolute difference between the 2011 and the 2001 *CII*_*rel*_, thus expressed as percentage-points; relative changes in *CII*_*rel*_ were obtained by dividing the absolute changes by the *CII*_*rel*_ in 2001.

Analyses were performed in Stata14, SAS 9.3, and in R 3.5.1 (R Core Team, 2018).

## Results

### Educational distribution

For both men and women, the educational composition of the population moved moderately upwards in 2011 compared to 2001, with fewer people in the low EL, and more people in the mid and high ELs (Table 1).

**Table 1:** Distribution of the educational attainment in the non-migrant population in the 2001 and 2011 censuses, population 25 years and older, Belgium

Educational level	Males		Females	
	*2001*	*2011*	*2001*	*2011*
Low	42.0%	36.3%	45.4%	38.2%
Mid	26.6%	32.5%	23.8%	28.7%
High	23.4%	26.5%	22.4%	28.4%
Missing	8.0%	4.7%	8.4%	4.7%

### Life and health expectancies in 2011

A low-mid-high gradient was observed in 2011 for the LE at each age (Table 2), and in both sexes. At age 25, the low-versus-high gap reached 6.07 years in men and 4.58 years in women. The absolute gap, although progressively decreasing with age, remained substantial until age 65. The gradient was more pronounced for DFLE_25_, with low-versus-high gaps reaching 10.47 and 13.44 years in men and women respectively.

**Table 2:** Life expectancy (LE) and disability-free LE (DFLE) at age 25, 30, 50 and 65 in Belgium by sex and educational attainment, calculated from the census 2011 with five years follow up, non-migrant population

Age	LE					DFLE				
	Low	Mid	High	Missing	Low-versus-High Gap	Low	Mid	High	Missing	Low-versus-High Gap
Male										
25	51.74	54.73	57.81	48.53	6.07	37.02	42.05	47.49	35.35	10.47
30	47.13	49.96	52.90	43.94	5.77	32.87	37.55	42.80	30.86	9.93
50	29.13	31.23	33.56	26.17	4.43	18.10	21.82	24.78	15.94	6.68
65	17.43	18.84	20.39	15.47	2.96	10.80	12.19	13.33	9.34	2.53
Female										
25	57.32	60.09	61.90	53.83	4.58	35.54	42.98	48.98	37.50	13.44
30	52.52	55.17	56.94	49.15	4.42	32.08	38.37	44.34	33.33	12.26
50	33.94	36.03	37.47	31.29	3.53	19.51	22.30	27.22	17.95	7.71
65	21.21	22.77	23.81	19.77	2.60	10.70	12.72	15.31	10.72	4.61

### Evolution of the LE_25_ and the LE_25_ inequalities between 2001 and 2011

Table 3 presents the absolute inequalities in LE_25_ for each EL compared to the highest EL in the 2001 census, 2011 census and the change observed between both censuses. The low-mid-high gradient for LE_25_ observed in the 2011 census was also present in 2001 (Table 3). The gradient changed however during the inter-census period.

**Table 3:** Life expectancy at age 25 (LE_25_), LE_25_ inequalities by educational level (gap) and Composite Inequality Indices (CII) of LE_25_ in the non-migrant population in 2001, 2011 and change over time (Δ), Belgium

Educational level	LE_25_, 2001	LE_25_, 2011	Δ LE_25_	*P* value	Ineq, 2001	Ineq, 2011	Change Ineq, abs	Change Ineq,%	*P* value
*Male*									
GAPS									
Low	50.63	51.74	1.11	< 0.001	5.19	6.07	+ 0.87	+ 16.8%	< 0.001
Mid	53.18	54.73	1.55	< 0.001	2.65	3.08	+ 0.43	+ 16.2%	< 0.001
High	55.82	57.81	1.99	< 0.001	ref	ref	ref	ref	/
CIIs									
CII, Abs.	.	.	.	.	3.14	3.36	+ 0.23	+ 7.3%	< 0.001
CII, Rel.	.	.	.	.	6.1%	6.3%	+ 0.2%	+3.3%	0.004
*Female*									
GAPS									
Low	57.14	57.32	0.18	< 0.001	3.76	4.58	+ 0.82	+ 21.8%	< 0.001
Mid	59.14	60.09	0.95	< 0.001	1.76	1.81	+ 0.05	+ 2.8%	0.4561
High	60.90	61.90	1.00	< 0.001	ref	ref	ref	ref	/
CIIs									
CII, Abs.	.	.	.	.	2.32	2.38	+ 0.05	+ 2.2%	0.217
CII, Rel.	.	.	.	.	4.1%	4.0%	− 0.1%	− 2.4%	0.696

In men, the LE_25_ increased in all three EL (Table 3), but more so among the highly educated. This resulted in a 16.8% increase of the low-versus-high difference between 2011 and 2001, raising from 5.19 in 2001 to 6.07 in 2011, or a 0.87-year increase of the gap. The *CII*_*abs*_ increased by 7% between the two periods, as did the *CII*_*rel*_ which increased by 0.2 percentage-points (2.8% relative increase).

Among women, LE_25_ was much higher than among men irrespective of EL, with gender gaps reaching 6.51, 5.96 and 5.08 years respectively in the low, mid and high EL in 2001 (Table 3). Compared to men, LE_25_ increased more moderately between the 2 censuses (column 3), leading in 2011 to a slight decrease in the gender gap to 5.58, 5.36 and 4.09 years in the low, mid and high EL respectively (column 2, difference in upper and lower part).

Inequalities in LE_25_ were smaller among women than among men in both censuses, but increased by 22% between the two censuses, from 3.8 in 2001 to 4.6 in 2011. The *CII*_*abs*_ and *CII*_*rel*_ in LE_25_ did not significantly change over time in women.

### Evolution of the DFLE_25_ and of the DFLE_25_ inequalities between 2001 and 2011

As for LE_25_, DFLE_25_ showed a low-mid-high gradient for both genders and in both censuses (Table 4).

**Table 4:** Disability-Free Life Expectancy at age 25 (DFLE_25_), DFLE_25_ inequalities by educational level (gap) and Composite Inequality Indices (CII) of DFLE_25_ in the non-migrant population in 2001, 2011 and change over time (Δ), Belgium

Educational level	DFLE_25_, 2001	DFLE_25_, 2011	Δ DFLE_25_	P value	Ineq, 2001	Ineq, 2011	Change Ineq, abs	Change Ineq, %	*P* value
*Male*									
GAPS									
Low	36.34	37.02	0.68	0.3994	6.51	10.47	+ 3.96	+ 61%	< 0.001
Mid	41.40	42.05	0.65	0.4056	1.45	5.44	+ 3.99	+ 275%	< 0.001
High	42.85	47.49	4.65	< 0.001	ref	ref	ref	ref	/
CIIs									
CII, Abs.	.	.	.	.	3.39	5.85	+ 2.46	+ 73%	0.001
CII, Rel.	.	.	.	.	8.7%	14.0%	+ 5.3%	+ 61%	0.003
*Female*									
GAPS									
Low	39.32	35.54	−3.78	< 0.001	9.30	13.44	+ 4.14	+ 45%	0.0042
Mid	43.37	42.98	−0.40	0.6733	5.24	6.00	+ 0.76	+ 15%	0.5979
High	48.62	48.98	0.36	0.7396	ref	ref	ref	ref	/
CIIs									
CII, Abs.	.	.	.	.	5.97	7.19	+ 1.22	+ 20%	0.191
CII, Rel.	.	.	.	.	14.3%	17.1%	+ 2.8%	+ 20%	0.148

Among men, the DFLE_25_ increased by 4.7 years in the highest EL, but did not change significantly in the other ELs, leading to an important increase of the inequalities between both censuses. The low-versus-high gap increased by almost 4 years (+ 61%), the *CII*_*abs*_ increased by 2.46 years, while the *CII*_*rel*_ increased by 5.3 percentage-points (61% relative increase) between 2001 and 2011.

Among women, the DFLE_25_ was higher in 2001 than among men independently of EL. The gender gap in DFLE was however more moderate than in LE_25_: 2.98, 1.97 and 5.77 among the low, mid and highly educated respectively (Table 4, column 2, difference between lower section and upper section). Between 2001 and 2011, the DFLE_25_ in women decreased considerably in the low EL (− 3.78 years), but stayed stable in the other EL. This led to a decrease in all EL-specific gender gaps, with even an inversion of the gender gap in the low EL group (respectively: − 1.48, 0.93 and 1.49 in low, mid, high EL).

This evolution of the DFLE_25_ in women also led to a large increase in the low-versus-high DFLE_25_ inequality, from 9.30 in 2001 to 13.44 years in 2011 (+ 4.1 years, or 45% increase). Both *CII*_*abs*_ and *CII*_*rel*_ were higher among women than among men and increased over time. Even though this change was quite large (2.8 percentage-points, corresponding to 20% relative increase for the *CII*_*rel*_), it was not statistically significant because of the large variance of the DFLEs.

## Discussion

### Summary of main findings

Our results show that the evolution of the inequalities between 2001 and 2011 differed for LE_25_ and DFLE_25_, and differed also by gender: the LE_25_ in men increased in all ELs, but faster among the tertiary educated, leading to an increase of all measures of inequalities (absolute LE_25_ gap, absolute and relative CIIs). Among women, the LE_25_ by EL increased less than in men, and even remained quasi stable in the low EL group. The low-versus-high LE_25_ gap increased, while the summary inequality measures persisted at the same level as in 2001.

An increase of the inequalities in DFLE_25_ was observed in both genders, but concomitant change in health outcomes differed by gender: in men the increase of the DFLE_25_ inequalities was associated with a substantial increase of the DFLE_25_ in highly educated men only. In women, the increase of the DFLE_25_ inequalities was combined with a strong deterioration of the DFLE_25_ in low educated women while remaining almost unchanged in the middle and high educated women.

### Comparison with other studies

Our results regarding the gap in LE_25_ are in line with the ones of Eggerickx [29], showing LE_25_ gaps of 7.3 and 6.4 years between low and high educated men and women. This study compared those without diploma with those with tertiary education, explaining the slightly higher LE_25_ gap compared to our study. Their main objective however was to assess inequality trends using a multidimensional score combining education, professional activity and housing. Relative socioeconomic classes were defined based on quartiles of this score. Although the authors recognize some limitations in the completeness of the data used in the score, this approach allows for trend analyses that are not hampered by changes in the size of the classes. They reported an increase in LE_25_ socioeconomic inequality between 2001 and 2011 in both sexes, while in our study this increase remained significant only in men after taking into account the shift in educational level. Our results contrast however with the ones of the OECD studies [5, 37], using unlinked data for Belgium. Even though the OECD studies considered the same EL groups as in our study, they found low-versus-high EL gaps of 9.8 and 6. 8 years for LE_25_ [5], or 9.7 and 6.7 years in LE_30_ [37] respectively in men and women, resulting in an implausibly unfavorable ranking of Belgium among OECD countries in terms of health inequalities. The impact of the numerator-denominator bias when using unlinked data has been emphasized in this OECD report, and particularly for Belgium. It was also previously studied at Belgian level and turned out to be extremely high [41]. With the use of linked data in our study, removing the numerator-denominator bias from the analysis, Belgium would rank lower than the male OECD25 average gap of 7.7 years and just above the female average gap of 4.2 years (Fig. 1).

In a systematic review of the life expectancy above 50 in Europe, Mosquera et al. found educational LE_50_ gaps in men ranging from 2.6 to 11.3 years, and gaps in women ranging from 1,6 to 6.9 years [3]. With LE_50_ gaps of respectively 4.4 and 3.5 years in men and women, Belgium is situated on an average position.

Reviews of studies analyzing inequality in health expectancy in Europe have pointed out their methodological heterogeneity, limiting the comparison of results [3, 42]. In a recent overview, Mosquera et al. reported gaps in DFLE_65_ ranging from 2.6 to 6.2 years in men and 2.3 to 6.3 years in women, but most of those results were based on quite old data (Share 1995–1997). Gaps in DFLE_65_ in Belgium in those previous results were respectively 2.9 and 2.3 in men and women, which placed Belgium at a very favorable position among the included countries; in our study DFLE_65_ gaps were 2.53 and 4.61. More recent results and trends in DFLE inequalities have only been published at national level. For instance, Danish studies [43–45] have found an increase in LE_30_ gaps over the past 25 years, with gaps reaching 6.4 years in men and 4.7 in women in 2011, results very similar to ours; gaps in DFLE and their changes were studied at ages 50 and 65. After the financial crisis, DFLE_50_ gaps increased in men but remained stable in women, with DFLE_65_ persisting at 2 years in men and 3 years in women.

### Strengths and limitations

One strength of the study is certainly the use of the census database covering the whole population and linking SE characteristics with mortality for the total population. This dataset is highly reliable with regards to mortality and does not suffer from a numerator-denominator bias [46–48], as would be the case with the use of the death certificates database, where socio-economic data are poorly recorded in Belgium. Information on EL was however collected differently in the two censuses. In 2001, data were collected through an individual questionnaire, while in 2011 the previously existing information was updated with administrative databases. This difference in data collection can lead to comparability issues for some SE variables, for instance the EL of newly graduates is probably more reliable in 2011 than it was in 2001. Of particular concern is the absence of information concerning the EL for all new migrants in the 2011 census, leading to an important proportion of missing values for the migrant population (34%). For this reason, we have limited our analyses to the non-migrant population. The proportion of missing values for the education variable in the non-migrant population was equal to 8.2% in 2001 and to 4.7% in 2011. In previous censuses, the non-response for EL was shown to be selective in at least two regards [24]: people with a low EL were less inclined to declare their EL, and very sick people were unlikely to complete a census form. In 2011, there was no form to fill in, so that the meaning and implications of missing information may differ between the two censuses. People with missing information for the education variable experienced a lower LE_25_ and DFLE_25_ than any other group. The missing-versus-high LE_25_ gap was equal to 11 years in 2001 and decreased to 9 years in 2011; the lower proportion of people with missing EL might suggest that the composition of this group changed over time [24]. We did not include them in the analysis, which is likely to induce a conservative bias, leading to some underestimation of inequalities [49]. To explore this issue, we performed an alternative scenario analysis pooling the missing category with the low EL. This indeed led to somewhat larger gaps and CIIs: for instance in 2011, the LE_25_ gap increased from 6.07 (missing ignored) to 6.45 years (missing pooled) in men and from 4.58 to 4.94 years in women (6 and 8% increase, respectively). Pooling the missing cases with the low category inversed the sense of change of the CII for LE_25_ (decreased instead of increasing) between 2001 and 2011, while no substantial difference was observed for the DFLE_25_ inequalities. Ignoring the missing cases probably leads to a slight underestimation of the absolute gaps, but an overestimation of the inequalities increase for LE_25_.

As health interview surveys only take place every 3 to 5 years in Belgium, we used data from the two HIS waves that were the closest to the follow up periods. The use of the HIS 2008 data can however be questionable, as this survey took place before the follow up period. However, as health status only slowly changes over time, we assume that this would have little impact on the results.

The concept of SE status is multifactorial, and different breakdown variables (like education, occupation, income, wealth, or a combination of those) can provide different results. In this study, we chose the EL as SE indicator. The EL has the advantage that it remains relatively stable over the life course from early adulthood onwards [22, 50]. The risk of ‘reverse causality’ is therefore weaker than for the association between health and income or occupation. However, education alone does not capture all aspects of SE inequalities.

The meaning of the EL varies for different birth cohorts, because of the upward shift of educational attainment. This shift causes the low EL group to be more homogenous and more negatively selected on the social scale than previously was the case. This is an important shortcoming when comparing social inequalities across birth cohorts, and not easy to solve. It was sometimes argued that the use of the relative inequality Index (RII) could solve the problem, but this is not the case: the RII uses relative positions of the SE categories on the social scale, but this repositioning, while adequately representing an additional dimension and its change, does however not adjust for it. This complicates the interpretation of trends. In our study, we used the CII to deal with this problem; this composite indicator weighs each EL according to its size, which is already part of the solution, but does not yet correct for the increased homogeneity of the lowest EL group. Only few studies have taken this problem into account and tried using an SE indicator of which the meaning remained stable over time [29, 51]. A Danish study addressed this issue by comparing LE inequalities evolution when using fixed versus relative educational classes and concluded that, the inequality rise persisted after having taken the EL shift into account [43].

We grouped the EL into three broad categories, which is in line with recent studies [5, 17]. Indeed, as the educational attainment has increased in most European countries, there are less individuals belonging to the categories ‘no education’ or ‘primary education’. This is to be accounted for when comparing with studies classifying EL in more or different categories [28, 29].

### Interpretation and policy implications

The improvement of LE_25_ in all ELs and both sexes is a clear public health progress. However, the increasing LE_25_ gap highlights that not all educational groups benefit equally from the LE improvement.

The considerable increase of DFLE_25_ in high educated men is certainly a progress; however, the stability of the DFLE_25_ in the other groups, again reflects a differential benefit across the ELs. The group of less educated women is the only one to experience a worsening of health between both periods. Since the improvement of the educational level has been particularly important in women, the group of women with the lowest educational level represents a diminishing group, that is probably worse off today than 10 years ago, and deserves further investigation and efforts.

Tackling health inequalities requires strategies encompassing multiple areas of intervention. Various sets of interventions have been implemented in some European countries, but their translation into results has not been fully analyzed [12]. In Belgium, although regional and federal commitments exist for addressing health inequalities, no comprehensive plan has been set up, but important efforts have been made to provide better access to universal health care and education. Research focusing on the causes of the inequalities in Belgium and on the health impact assessment of different strategies would help in the elaboration of this plan. As a first step to inform where to prioritize, a previous study [27] has shown that lung cancer, ischemic heart disease (IHD), chronic obstructive pulmonary disease (COPD) and suicide in men, and IHD, stroke, lung cancer and COPD in women had the highest impact on mortality inequalities, indicating an important place for tobacco-related diseases in mortality inequalities. This is corroborated by the Belgian HIS, indicating increasing inequalities in smoking behaviour; the reduction of smoking in low educated people should thus take a prominent place in a national plan tackling inequalities. Mackenbach et al. [12] cite strategies combining nicotine replacement and various approaches of cessation support as effective in some contexts.

Another important evolution is the increase of the share of highly educated people, which is indubitably a progress, not only because it results in more people being in good health, but also because polices aimed at tackling health inequalities have to address social conditions contributing to unequal chances in health [7, 24]. Improving the educational attainment is a key strategy to achieve this goal [6]. Actually, the improvement of the educational attainment should result in healthier life styles, access to a better work situation and a better living environment. The upwards shift of the educational attainment, by altering the composition of the groups, complicates the interpretation of the evolution of inequalities over time. It is noteworthy that the upwards shift in educational attainment has not had a dilution effect on the health outcomes: although the share of the highest educated increases, they continue to be in better health than the other ELs. The composite inequality indices, that weigh for the composition of the population, have slightly increased among men for LE, and remained stable in women. For DFLE, the composite indices increased more than for LE, and more in men.

## Conclusion

With this study, we monitor the evolution of the inequalities in LE_25_ and DFLE_25_ after the turn of the century. The assessment of the inequality trend is complex. On the one hand, policies promoting the educational attainment have shifted the educational distribution upwards and the LE_25_ increased in all ELs, which is a valuable public health outcome. However, the LE_25_ increased faster in people with high EL, leading to an increase in LE_25_ differentials in both sexes. After taking into account the upwards shift of the educational distribution, the summary inequality index increased only for men. The DFLE_25_ increased only in highly educated men, and decreased in low educated women, leading to an increase of inequalities in both genders. A general plan to tackle health inequality should be set up, with particular efforts to improve the health of the low educated women.

## Additional file

Additional file 1: Appendix **Table S1.** Sensitivity analysis, LE_25_ inequalities. Scenario = missing for Educational level (EL) grouped with low EL. People of Belgian nationality, 2001, 2011 and change. (DOCX 23 kb)

## Abbreviations

*CII *_*abs*_: Composite Inequality Index, absolute
*CII *_*rel*_: Composite Inequality Index, relative
DFLE: Disability-Free Life Expectancy
DFLE_25_: Disability-Free Life Expectancy at age 25
EL: Educational Level
LE: Life Expectancy
LE_25_: Life Expectancy at age 25
SE: Socio-Economic

## References

[1] Feinstein JS (1993). The relationship between socioeconomic status and health : a review of the literature. The Milkbank Quarterly.

[2] Mackenbach J. Health inequalities: Europe in profile. Expert report commissionned by the EU. Department of health publications; 2006.

[3] Mosquera I, Gonzalez-Rabago Y, Martin U, Bagigalupe A. Review of socio-economic inequalities in life expectancy and health expectancy in Europe. Factage project - WP2; 2018.

[4] Majer IM, Nusselder WJ, Mackenbach JP, Kunst AE (2011). Socioeconomic inequalities in life and health expectancies around official retirement age in 10 Western-European countries. J Epidemiol Community Health.

[5] Murtin F, Mackenbach J, Jasilionis D, Mira d’Ercole M (2017). Inequalities by education in OECD coutries: insights from new OECD estimates.

[6] WHO Regional Office for Europe (1999). Health 21: the health for all policy framework for the WHO European region.

[7] Marmot M, Friel S, Bell R, Houweling TA, Taylor S (2008). Closing the gap in a generation: health equity through action on the social determinants of health. Lancet.

[8] Executive Agency for Health and Consumer (2007). Second Programme of Community Action in the Field of Health 2008-201e3. European Commission.

[9] Vlaamse overheid (2017). Vlaamse Actieplan Geestelijke Gezondheid, Strategisch plan 2017–2019.

[10] Gouvernement wallon (2017). Plan prévention et promotion de la santé en Wallonie.

[11] Arrêté royal du 18 juillet 2013 portant fixation de la vision stratégique fédérale à long terme de développement durable: http://www.etaamb.be/fr/arrete-royal-du-18-juillet-2013_n2013011468.html. Moniteur Belge 2013 Oct 8. Accessed Aug 2018.

[12] Mackenbach JP, Bakker MJ (2003). Tackling socioeconomic inequalities in health: analysis of European experiences. Lancet.

[13] Dahlgren G, Whitehead M (2000). Policies and strategies to promote equity in health. *.

[14] Braveman PA (2003). Monitoring equity in health and healthcare: a conceptual framework. J Health Popul Nutr.

[15] Mackenbach JP (2012). The persistence of health inequalities in modern welfare states: the explanation of a paradox. Soc Sci Med.

[16] Harper S, King NB, Meersman SC, Reichman ME, Breen N, Lynch J (2010). Implicit value judgments in the measurement of health inequalities. Milbank Q.

[17] Mackenbach JP, Kulhanova I, Menvielle G, Bopp M, Borrell C, Costa G (2015). Trends in inequalities in premature mortality: a study of 3.2 million deaths in 13 European countries. J Epidemiol Community Health.

[18] Speybroeck N, Harper S, de Savigny D, Victora C (2012). Inequalities of health indicators for policy makers: six hints. International Journal of Public Health.

[19] Mackenbach JP, Kunst AE (1997). Measuring the magnitude of socio-economic inequalities in health: an overview of available measures illustrated with two examples from Europe. Soc Sci Med.

[20] Wagstaff A, Paci P, van Doorslaer E (1991). On the measurement of inequalities in health. Soc Sci Med.

[21] Harper S, Lynch J, Meersman SC, Breen N, Davis WW, Reichman ME (2008). An overview of methods for monitoring social disparities in cancer with an example using trends in lung cancer incidence by area-socioeconomic position and race-ethnicity, 1992-2004. Am J Epidemiol.

[22] Bostrom G, Rosen M (2003). Measuring social inequalities in health—politics or science?. Scand J Public Health.

[23] Whitehead M, Dahlgren G (2006). Levelling up (part 1) : a discussion paper on concepts and principles for tackling social inequities in health.

[24] Deboosere P, Gadeyne S, Van Oyen H (2008). The 1991–2004 evolution in life expectancy by educational level in Belgium based on linked census and population register data. Eur J Popul.

[25] Gadeyne S (2006). The ultimate inequality : socio-economic differences in all-cause and cause-specific mortality in Belgium on the first part of the 1990s.

[26] Bossuyt N, Gadeyne S, Deboosere P, Van Oyen H (2004). Socio-economic inequalities in health expectancy in Belgium. Public Health.

[27] Renard F, Gadeyne S, Devleesschauwer B, Tafforeau J, Deboosere P (2017). Trends in educational inequalities in premature mortality in Belgium between the 1990s and the 2000s: the contribution of specific causes of death. J Epidemiol Community Health.

[28] Van Oyen H, Charafeddine R, Deboosere P, Cox B, Lorant V, Nusselder W (2011). Contribution of mortality and disability to the secular trend in health inequality at the turn of century in Belgium. Eur J Publ Health.

[29] Eggerickx T, Sanderson J, Vanderschrick C. Les inégalités sociales et spatiales de mortalité en Belgique: 1991–2016: Espace Population et Société; 2018. 10.4000/eps.7416.

[30] Deboosere P, Gadeyne S (1999). De Nationale Databank Mortaliteit. Aanmaak van een databank voor onderzoek van differentiële sterfte naar socio-economische status en leefvorm.

[31] Demarest S, Van der Heyden J, Charafeddine R, Drieskens S, Gisle L, Tafforeau J (2013). Methodological basics and evolution of the Belgian health interview survey 1997-2008. Archives of Public Health.

[32] Robine JM, Jagger C, Van Oyen H, Cambois E, Doblhammer G, Nusselder W (2010). The Minimum European Health Module.

[33] Van Oyen H, Bogaert P, Yokota RTC, Berger N (2018). Measuring disability: a systematic review of the validity and reliability of the global activity limitations Indicator (GALI). Arch Public Health.

[34] Eurostat, statistics explained. Health Life years statistics. 2017. https://ec.europa.eu/eurostat/statistics-explained/index.php/Healthy_life_years_statistics. Accessed Aug 2018.

[35] UNESCO (1997). International standard classification of education, ISCED 1997.

[36] Statbel. Historique du recensement de la population et des logements. 2018. https://statbel.fgov.be/fr/propos-de-statbel/que-faisons-nous/recensement-census. Accessed Aug 2018.

[37] OECD. Health at a Glance 2017: OECD Indicators. Paris: OECD Publishing; 2017. 10.1787/health_glance-2017-en.

[38] Nitika MSS, Lohani P (2017). Lexis expansion: a prerequisite for analyzing time changing variables in a cohort study. Nepal J Epidemiol.

[39] Jagger C, Van Oyen H, Robine JM. Health expectancy calculation by the Sullivan Method: A Practical Guide. 4th edition. Montpellier: ELHEIS; 2014. http://www.eurohex.eu/pdf/Sullivan_guide_pre%20final_oct%202014.pdf.

[40] Robert C, Casella G (2004). Monte Carlo statistical methods.

[41] Charafeddine R, Gadeyne S, Deboosere P, Berger N, Demarest S, Van Oyen H (2011). Social inequalities in healthy life expectancy. Alternative methods of estimation in the absence of the national census.

[42] Pongiglione B, De Stavola BL, Ploubidis GB (2015). A systematic literature review of studies analyzing inequalities in health expectancy among the older population. PLoS One.

[43] Bronnum-Hansen H, Baadsgaard M (2012). Widening social inequality in life expectancy in Denmark. A register-based study on social composition and mortality trends for the Danish population. BMC Public Health.

[44] Bronnum-Hansen H, Eriksen ML, Andersen-Ranberg K, Jeune B (2017). Persistent social inequality in life expectancy and disability-free life expectancy: outlook for a differential pension age in Denmark?. Scand J Public Health.

[45] Bronnum-Hansen H, Baadsgaard M, Eriksen ML, Andersen-Ranberg K, Jeune B (2015). Educational inequalities in health expectancy during the financial crisis in Denmark. Int J Public Health.

[46] Williams GM, Najman JM, Clavarino A (2006). Correcting for numerator/denominator bias when assessing changing inequalities in occupational class mortality, Australia 1981 -2002. Bull World Health Organ.

[47] Blakely T, Robson B, Atkinson J, Sporle A, Kiro C (2002). Unlocking the numerator-denominator bias. I: adjustments ratios by ethnicity for 1991-94 mortality data. The New Zealand census-mortality study. N Z Med J.

[48] Rey G, Rican S, Luce D, Menvielle G, Jougla E (2013). Measuring social inequalities in cause-specific mortality in France: comparison between linked and unlinked approaches. Rev Epidemiol Sante Publique.

[49] Vanthomme K, Vandenheede H, Hagedoorn P, Gadeyne S (2017). Evolution of educational inequalities in site-specific cancer mortality among Belgian men between the 1990s and 2000s using a “fundamental cause” perspective. BMC Cancer.

[50] Galobardes B, Shaw M, Lawlor DA, Lynch JW, Davey SG (2006). Indicators of socioeconomic position (part 1). J Epidemiol Community Health.

[51] Beebe-Dimmer J, Lynch JW, Turrell G, Lustgarten S, Raghunathan T, Kaplan GA (2004). Childhood and adult socioeconomic conditions and 31-year mortality risk in women. Am J Epidemiol.

