# Supplementary material for: Evolution of educational inequalities in life and health expectancies at 25 years in Belgium between 2001 and 2011: a census-based study
Source: Arch Public Health. 2019 Feb 14;77:6. doi: 10.1186/s13690-019-0330-8 (PMC6376710; doi:10.1186/s13690-019-0330-8)
Supplement: Supplementary file 1 — Appendix Table S1. Sensitivity analysis, LE25 inequalities. Scenario = missing for Educational level (EL) grouped with low EL. People of Belgian nationality, 2001, 2011 and change. (DOCX 23 kb) [file 13690_2019_330_MOESM1_ESM.docx]

Appendix Table 1: Sensitivity analysis , LE_25_ inequalities . Scenario= missing for Educational level (EL) grouped with low EL;

People of Belgian nationality, 2001, 2011 and change

| *Males, LE_25_* |  |  |  |  |  |
| --- | --- | --- | --- | --- | --- |
|  |  |  |  |  |  |
| *Educational level* | *Ineq 2001* | *Ineq 2011* | *Change ineq* | *% change ineq* | *pvalue* |
| *Gaps* |  |  |  |  |  |
| *Low + missing* | 6.35 | 6.45 | 0.1 | 2% | 0.112 |
| *Mid* | 2.65 | 3.08 | 0.43 | 16% | <0.001 |
| *High* | 0 | 0 | 0 |  | / |
| *CIIs* |  |  |  |  |  |
| *CII, Abs.* | 3.88 | 3.65 | -0.23 | -6% | <0.001 |
| *CII, Rel.* | 0.08 | 0.07 | -0.1 | -13% | <0.001 |
|  |  |  |  |  |  |
|  |  |  |  |  |  |
| *Females,LE_25_* |  |  |  |  |  |
|  |  |  |  |  |  |
| *Educational level* | *Ineq 2001* | *Ineq 2011* | *Change ineq* | *% change ineq* | *pvalue* |
| *Gaps* |  |  |  |  |  |
| *Low+missing* | 5.03 | 4.94 | -0.1 | -2% | 0.1513 |
| *Mid* | 1.76 | 1.81 | 0.05 | 3% | 0.4561 |
| *High* | 0 | 0 | 0 |  | / |
| *CIIs* |  |  |  |  |  |
| *CII, Abs.* | 3.13 | 2.64 | -0.49 | -16% | <0.001 |
| *CII, Rel.* | 0.05 | 0.04 | -0.18 | -20% | <0.001 |
|  |  |  |  |  |  |
|  |  |  |  |  |  |
|  |  |  |  |  |  |

Appendix Table 2: Sensitivity analysis ,DFLE_25_ inequalities . Scenario= missing for Educational level (EL) grouped with low EL

People of Belgian nationality, 2001, 2011 and change

| *Males, DFLE_25_* |  |  |  |  |  |
| --- | --- | --- | --- | --- | --- |
| *Educational level* | *Ineq 2001* | *Ineq 2011* | *Change ineq* | *% change ineq* | *pvalue* |
| *Gaps* |  |  |  |  |  |
| *Low+missing* | 7.21 | 10.51 | 3.31 | 46% | 0.0032 |
| *Mid* | 1.45 | 5.44 | 3.99 | 275% | <0.001 |
| *High* | 0 | 0 | 0 |  | / |
| *CIIs* |  |  |  |  |  |
| *CII, Abs.* | 3.99 | 6.08 | 2.1 | 53% | 0.005 |
| *CII, Rel.* | 0.1 | 0.15 | 0.43 | 50% | 0.019 |
|  |  |  |  |  |  |
|  |  |  |  |  |  |
|  |  |  |  |  |  |
|  |  |  |  |  |  |
|  |  |  |  |  |  |
|  |  |  |  |  |  |
| *Females, DFLE_25_* |  |  |  |  |  |
| *Educational level* | *Ineq 2001* | *Ineq 2011* | *Change ineq* | *% change ineq* | *pvalue* |
| *Gaps* |  |  |  |  |  |
| *Low+missing* | 9.33 | 12.59 | 3.26 | 35% | 0.0161 |
| *Mid* | 5.24 | 6 | 0.76 | 15% | 0.5979 |
| *High* | 0 | 0 | 0 |  | / |
| *CIIs* |  |  |  |  |  |
| *CII, Abs.* | 6.27 | 7.12 | 0.85 | 14% | 0.359 |
| *CII, Rel.* | 0.15 | 0.17 | 0.12 | 13% | 0.316 |
